# Focal adhesion kinase FAK interplays with ROR1 in the aggressiveness of chronic lymphocytic leukemia

**DOI:** 10.1186/s12916-026-05025-1

**Published:** 2026-06-27

**Authors:** Maria Castronuovo, Guido Capasso, Allison Beltrame, Nayla Mouawad, Vincenzo Gramegna, Paolo Fantato, Elisa Pagnin, Francesco Angotzi, Alessandro Cellini, Andrea Serafin, Monica Facco, Andrea Visentin, Livio Trentin, Federica Frezzato

**Affiliations:** Hematology Unit, Department of Medicine, University of Padua, Padua, Italy

**Keywords:** Chronic Lymphocytic Leukemia, Focal Adhesion Kinase, ROR1

## Abstract

**Background:**

In chronic lymphocytic leukemia (CLL), B-cell receptor, chemokine receptor and integrin signaling contribute to disease progression by influencing cell survival, migration and microenvironment interactions. Focal adhesion kinase (FAK) has recently been involved in these disease processes. Our recent research revealed a correlation between activated FAK and molecules involved in CLL aggressiveness, particularly in IGHV-unmutated cases, including the Lyn kinase substrates HS1 and cortactin. In this context, ROR1 is a key player, sharing connections with Lyn substrates and FAK pathways.

**Methods:**

We assessed FAK and ROR1 bidirectional association by stimulating CLL cells with Wnt5a and measuring FAK phosphorylation by Western blot, while ROR1 expression was evaluated following FAK inhibitor treatment. Flow cytometry using CXCR4 and CD5 markers was performed to identify and sort proliferating (CXCR4^dim^/CD5^bright^) versus quiescent (CXCR4^bright^/CD5^dim^) B-cell subpopulations from CLL patients. FAK and ROR1 levels were compared between these fractions. Findings were further supported ex vivo in ibrutinib-treated patients. The FAK inhibitor defactinib was tested alone and in combination with BTK inhibitors on primary CLL cells cultured with or without stromal support, measuring apoptosis by Annexin V/PI staining.

**Results:**

We demonstrated that ROR1 triggering by Wnt5a increases FAK activation, while FAK inhibition reduces ROR1 expression. Significantly higher levels of FAK and ROR1 were detected in the proliferating subpopulation corresponding to cells egressing lymph nodes compared to quiescent cells. Ex vivo experiments confirmed high FAK and ROR1 levels in circulating lymphocytes redistributing from secondary lymphoid organs. Defactinib significantly enhanced apoptosis in CLL cells when combined with BTK inhibitors, even in supportive microenvironments, showing significant efficacy also against aggressive CLLs.

**Conclusions:**

Our findings reveal a reciprocal regulatory relationship between FAK and ROR1, with both proteins enriched in CLL cells that have exited the lymph nodes. Given defactinib’s favorable safety profile in solid tumor trials, combining FAK inhibition with BTK inhibitors could enhance therapeutic efficacy by limiting leukemic clone adaptability and addressing resistance and relapse. While anti-ROR1 therapies showed limited efficacy as monotherapy in CLL trials, targeting multiple nodes of this regulatory network may prove more effective.

**Supplementary Information:**

The online version contains supplementary material available at https://doi.org/10.1186/s12916-026-05025-1.

## Background

Chronic Lymphocytic Leukemia (CLL) is a lymphoproliferative disorder characterized by the accumulation of CD19+/CD5+/CD23 + clonal B cells in peripheral blood, bone marrow and secondary lymphoid organs (SLO). Its course varies from indolent to aggressive, emphasizing the importance of identifying new disease characteristics for prognostic and therapeutic purposes[1]. CLL pathogenesis involves deregulated apoptosis and impaired B-cell antigen receptor (BCR) signaling [2]. Additionally, CLL cells heavily depend on their microenvironment for survival and proliferation [3, 4]. The recirculation of CLL cells from blood to lymph nodes is therefore a key process in supporting leukemic cell survival. Critical signaling pathways, particularly the CXCR4/CXCL12 axis, facilitate the migration of CLL cells to these protective niches [5]. Adhesion molecules such as CD49d and CD62L enhance their retention within tissues, [6, 7] while S1P favors their egress from SLO. These complex interactions within the microenvironment play a crucial role in sustaining the disease and in potentially hindering therapeutic efficacy. The introduction of Bruton’s tyrosine kinase inhibitors (BTKi) has significantly improved the management of CLL. During treatment, a significant proportion of patients experience transient lymphocytosis, an increase in CLL cells within the peripheral blood, resulting from the mobilization of leukemic cells from SLO into the blood [8]. This highlights the complex interplay between CLL cells and their protective microenvironments.

While BTKi therapy has shown remarkable efficacy, this latter does not lack challenges. The emergence of resistance to BTKi and/or upregulation of alternative pathways that promote cell survival and migration may result in adverse clinical outcomes [9]. These adaptations often involve mechanisms related to the tumor microenvironment. Consequently, ongoing research is focused on identifying new therapeutic targets, particularly those involving the neoplastic microenvironment and leukemic B lymphocytes. Among these, focal adhesion kinase (FAK), a non-receptor tyrosine kinase, has emerged as a critical player in mediating extra- to intra-cellular signaling [10]. FAK contributes significantly to the survival and proliferation of leukemic cells through the activation of key downstream signaling pathways, such as the Phosphoinositide 3-kinase (PI3K)/Akt pathway and the Mitogen-Activated Protein Kinase (MAPK)/Extracellular Signal-Regulated Kinase (ERK) pathways [11]. The inhibition of kinase activity of FAK has been shown to induce apoptosis in leukemia cells by an inhibitor such as defactinib [12, 13]. This drug is being evaluated in phase II clinical trials for the treatment of solid tumors and its potential role in the treatment of onco-hematological diseases, including CLL, is being studied. A recent study has underscored the critical role of FAK in the migration of CLL cells, emphasizing FAK as a promising therapeutic target to inhibit CLL trafficking to protective lymphoid niches [14]. Moreover, FAK inhibition with defactinib in primary samples of CLL cells has shown a significantly reduction of CXCL12-induced migration and invasion in vitro [13]. We previously demonstrated that in CLL cells, particularly in poor-prognosis IGHV-unmutated patients, BCR triggering leads to FAK cleavage and phosphorylation, resulting in an active form of FAK. We also observed that two proteins previously described also by our group, named HS1 and cortactin [15–17], were overexpressed in that cohort and down-regulated by defactinib treatment. These findings suggest that the malignant phenotype in unfavorable-prognosis CLL patients may be driven by the overexpression of cortactin and HS1, which, along with the cleaved and phosphorylated active form of FAK, potentially constitute a druggable pathogenetic pathway in CLL [13]. In addition, recent studies have highlighted the role of ROR1 (Receptor Tyrosine Kinase-Like Orphan Receptor 1) [18] in CLL pathogenesis. Interestingly, ROR1 has been shown to interact with both HS1 and cortactin [19, 20], suggesting a complex interplay between these proteins in the regulation of CLL cell survival and migration. ROR1, like FAK, HS1, and cortactin, is involved in cytoskeletal reorganization and cell motility, processes crucial for CLL cell trafficking and homing to protective niches. Highly expressed ROR1 correlates with accelerated disease progression and poorer prognosis [21, 22] and several therapies targeting ROR1 are currently in development [18]. Recent evidence suggests a significant crosstalk between BCR and ROR1 pathways [23, 24]. Understanding the molecular mechanisms underlying this interaction could have direct therapeutic implications. Notably, FAK looks like as a potential converging point, potentially regulating migration and survival signals downstream of both BCR and ROR1 pathways [25]. Based on this background, we investigated FAK activation in CLL and its association with a disease-promoting phenotype. We explored the ROR1-FAK interplay by examining ROR1-mediated FAK activation and defactinib’s impact on ROR1 expression and signaling. Additionally, we evaluated defactinib’s effects on CLL cells, both alone and in combination with BTKi, in the presence and absence of HS5 stromal cells, known to promote CLL survival and drug resistance. Our findings highlight the FAK-ROR1 interaction as a promising therapeutic target, particularly for patients resistant to current treatments.

## Methods

### Patients, cell separation and culture conditions

Leukemic B cells were obtained from untreated CLL patients, enrolled by the Hematology Unit of Azienda Ospedale-Università Padova, satisfying standard criteria for CLL. Written informed consent according to the Declaration of Helsinki and ethical approval were obtained. Immunophenotypic analysis of peripheral blood lymphocytes was performed by flow cytometry. Surface antigens were detected using fluorochrome-conjugated monoclonal antibodies. Forward scatter (FSC) and side scatter (SSC) parameters were used to identify lymphocytes within the peripheral blood cell population. Cells within the lymphocyte gate were analyzed with fluorescent antibodies to determine the percentage of CD19^+^/CD5^+^ cells. Peripheral blood mononuclear cells (PBMCs) were isolated by density-gradient centrifugation with Lymphoprep (Stem Cell Technologies Inc.; Vancouver, Canada). When the leukemic B-cell fraction was < 90%, additional enrichment was performed using the Human B Cell Enrichment Cocktail (Stem Cell Technologies Inc.). The resulting leukemic B-cell population consistently exhibited ≥ 95% purity (CD19^+^/CD5^+^), as assessed by flow cytometry [26]. In other set of experiments, cells were obtained from CLL patients undergoing Ibrutinib-containing regimen, at different time points. Cells were cultured in RPMI 1640 medium (Thermo Fisher Scientific; MA, USA) with 2% FBS and 5% Penicillin/Streptomycin (Euroclone S.p.A.; Milan, Italy) at 2 × 10^6^/mL and incubated at 37 °C in a humidified atmosphere containing 5% CO_2_ with or without: 5µM defactinib and/or 5µM ibrutinib, 5µM acalabrutinib, and 5µM zanubrutinib (Selleckchem; TX, USA) for 24–48 h. HS-5 cell line was also employed, which represent a component of the marrow microenvironment, as previously detailed [4]. For other experiments, Wnt5a and CXCL12 (R&D Systems; MN, USA) were used at 200ng/mL concentration. All patients included in this study are detailed in Additional file 1: Table S1.

### Flow cytometry

Apoptosis was assessed using the Annexin V Apoptosis Detection Kit (Immunostep; Salamanca, Spain). Aliquots of 2.5 × 10^5^ cells were harvested, washed, and resuspended in 100µL of binding buffer (a Ca^2+^-rich solution that promotes Annexin V binding to phosphatidylserine). Subsequently, 3µL of Annexin V-FITC were added, and samples were incubated for 10 min at room temperature in the dark. After incubation, 100µL of binding buffer and 3µL of propidium iodide-PE (provided in the kit) were added, and cells were immediately analyzed by flow cytometry. For each sample, 20,000 events were acquired, and cell viability was expressed as the percentage of Annexin V/PI double-negative cells relative to the total population analyzed. For FAK expression, 1 × 10^6^ B lymphocytes per tube were stained with surface monoclonal antibodies anti-CD19-PeCy7 (BD Biosciences; NJ, USA), anti-CD5-FITC (Abcam; Cambridge, UK), and anti-CXCR4-PE (R&D Systems) for10 min at room temperature. Since FAK is an intracellular protein, cells were subsequently washed, fixed, and permeabilized using the FIX & PERM™ Cell Permeabilization Kit (Thermo Fisher Scientific), according to the manufacturer’s instructions. During the permeabilization step, one tube was incubated with anti-FAK-Alexa Fluor^®^ 405 (Abcam) for 10 min at room temperature, while the parallel tube without this antibody was used as the Fluorescence Minus One (FMO) control. ROR1 expression was assessed using the same staining procedure, but without fixation and permeabilization, as ROR1 is an extracellular antigen. In this case, anti-ROR1-APC (Miltenyi Biotech; Bergisch Gladbach, Germany) was used. After a final wash, 100,000 events per sample were acquired by flow cytometry. Samples were first gated on intact cells based on FSC vs. SSC parameters. A second gating step was then applied on CD19^+^ cells, which were further analyzed in a density plot of CD5 vs. CXCR4 expression to distinguish the CD5^dim^/CXCR4^bright^ and CD5^bright^/CXCR4^dim^ subpopulations, allowing evaluation of FAK and ROR1 expression within these two fractions.

### Cell sorting and protein expression analysis

Leukemic B cells were isolated from peripheral blood of CLL patients by enrichment with the Human B Cell Enrichment Cocktail, followed by density-gradient centrifugation with Lymphoprep (Stem Cell Technologies Inc.). Cells were then stained with fluorochrome-conjugated antibodies against CXCR4 and CD5, as described above, to allow discrimination of proliferating (CD5^dim^/CXCR4^bright^) and quiescent (CD5^bright^/CXCR4^dim^) subpopulations. Cell sorting was performed using a FACSAria (BD Biosciences) cell sorter, and the two populations were collected separately. Sorted cells were lysed, and protein lysates were subjected to western blot analysis. Immunoblotting was performed using the following primary antibodies: anti-FAK (Merck KGaA; Darmstadt, Germany), anti-FAK-Y397, and anti-ROR1 (Cell Signaling Technology CST; MA, USA).

### Western blotting analysis

Samples were prepared as previously described [27]. Briefly, cells (5 × 10^5^ per assay) were lysed in buffer containing 20mmol/L Tris, 150mmol/L NaCl, 2mmol/L EDTA, 2mmol/L EGTA, and 0.5% Triton X-100, supplemented with a complete protease inhibitor cocktail (Roche; Mannheim, Germany) and 1mmol/L sodium orthovanadate (Calbiochem; NJ, USA). Proteins were separated by SDS-PAGE on 10% gels, transferred onto PVDF membranes, and immunostained with the following primary antibodies: anti-FAK (Merck KGaA), anti-FAK-Y397, anti-PARP, anti-ROR1, anti-Smac/DIABLO (Cell Signaling), anti-Annexin II (BD Biosciences), anti-HSP70, anti-GAPDH (Abcam), and anti-β-actin (Merck KGaA). For phosphorylated FAK detection, we quantified the 50 kDa band, representing a cleaved/activated form of the protein, due to its consistent signal and superior signal-to-noise.

### Cell Migration Assay

Cell migration was assessed using 3 μm-pore polycarbonate transwell inserts in 24-well plates (Corning; NY, USA). Briefly, 2 × 10^6^ cells were incubated in 1mL RPMI medium with or without 5µM Defactinib for 6–18 h at 37 °C. Cells were then stimulated or not with 200 ng/mL Wnt5a (R&D Systems). A total of 1 × 10^6^ cells in 100µL RPMI were transferred to the top chambers of the transwell inserts, while the lower chambers contained 600µL RPMI medium with 200ng/mL CXCL12 (R&D Systems). Cells were allowed to migrate for 3 h at 37 °C, and migrated cells were collected and counted using a FACSCanto (BD Biosciences) II for 60 s in duplicates.

### RPPA analysis

The procedure, samples and antibodies used have been previously detailed [28]. Briefly, cells were lysed and protein concentration was determined by the Bradford method. Lysates were diluted to ≤1 mg/mL in Novex Tris-glycine SDS sample buffer (Thermo Fisher Scientific) supplemented with 2-mercaptoethanol, stored at − 80 °C, and boiled for 8 min immediately before use. Samples were then loaded into 384-well plates and serially diluted with lysis buffer to generate four-point dilution curves. Commercial cell-line lysates were included as positive controls. Samples were printed in duplicate onto nitrocellulose-coated glass slides (FAST slides, Cytiva; MA, USA). Arrays were blocked for 1 h at room temperature in blocking solution (2 g I-Block, Tropix and 0.1% Tween-20 in 1 L PBS 1×) prior to antibody staining. Staining was performed on an automated slide stainer (Agilent Technologies; CA, USA) using the CSA kit (Agilent Technologies). Slides were scanned at 600dpi with an Epson Perfection V350 scanner (Seiko Epson Corporation; Suwa, Japan), and numeric intensity values were extracted from array images using Microvigene Software (VigeneTech Inc.; MA, USA).

### Statistical analysis

Statistical analyses were performed using Prism 9 (GraphPad Software Inc.; CA, USA). Prior to applying statistical tests, data were assessed for normality using the Shapiro-Wilk test. Based on these results, appropriate parametric or non-parametric tests were chosen. P values < 0.05 were considered significant.

Detailed protocols for co-immunoprecipitation and ImageStream [29–31] analyses are provided in Additional file 1: Supplementary Methods.

## Results

### A disease-promoting profile is highlighted in patients with activated/phosphorylated FAK

**Fig. 1:**
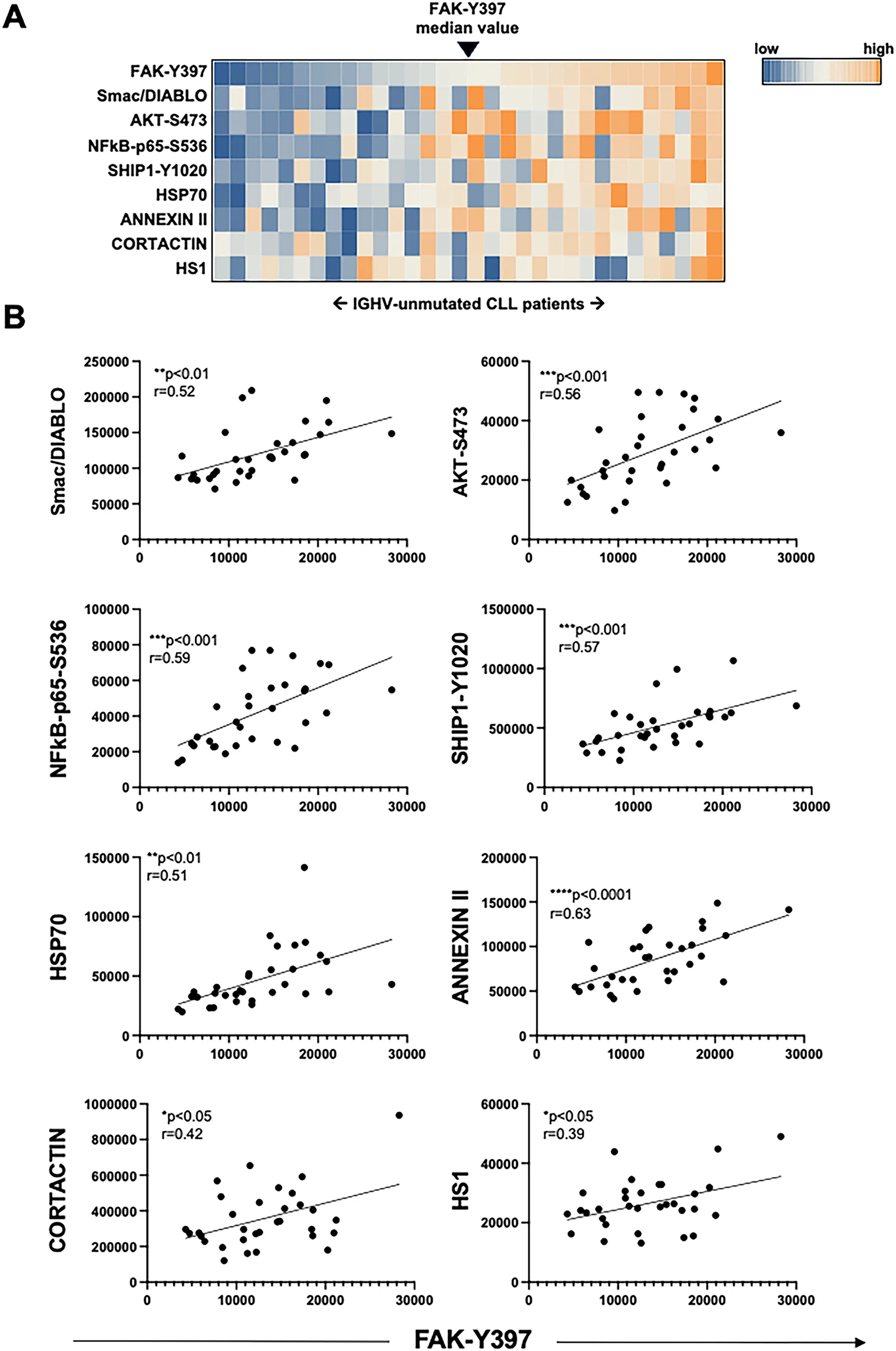
Correlations of FAK-Y397 phosphorylation with protein expression in IGHV-unmutated CLL patients.** (A)** Heatmap representing the expression levels of the analyzed proteins in relation to FAK phosphorylation at Y397, as assessed by Reverse Phase Protein Array [28] List of proteins analyzed is reported on the left. **(B)** Proteins showing a significant correlation with FAK-Y397 phosphorylation, determined by Pearson’s correlation (*n* = 32)

We re-analyzed the results of a previous Reverse Phase Protein Array (RPPA) study [28] according to the level of FAK phosphorylation at the activatory Y397 site, thus observing that different proteins were significantly overexpressed/hyperactivated in the phospho-FAK-high group as compared to samples with low phospho-FAK. A strong correlation was observed between FAK-Y397 and several proteins: Smac/DIABLO, AKT-Ser473, NF-κB-Ser536, SHIP-1-Y1020, HSP70, and ANNEXIN II. Additionally, this correlation was confirmed for cortactin and HS1, consistent with our previous findings [13]. These correlations were stronger in IGHV-unmutated patients compared to the mutated ones. The heat map in Fig. 1A represents the expression of all the identified proteins vs. FAK-Y397, and Fig. 1B shows the correlation with FAK-Y397 using the Pearson method in IGHV-unmutated patients. Protein amount has also been analyzed in FAK-Y397^low^ and FAK-Y397^high^ samples, using as cut-off the median value of FAK-Y397 (Additional file 1: Fig. S1). These new findings align with and extend our previous observations, namely that IGHV-unmutated poor-prognosis CLL patients present a higher amount of the cleaved/activated form of FAK protein, which is also phosphorylated at Y397; these features contribute to a more aggressive behavior of the disease [13]. The current results provide a more comprehensive picture of the molecular landscape associated with FAK activation in CLL, particularly in the context of IGHV mutational status.

The patient cohort herein analyzed was split according to cytogenetic analysis by FISH (13q, normal karyotype, 11q- and 17p-, Additional file 1: Fig. S2A-B) demonstrating a higher amount of activated FAK in 11q- patients where 11q deletion identifies a clinical subset of CLL characterized by extensive lymph node involvement.

### FAK activation correlates with ROR1 expression and it is enhanced by ROR1 stimulation in CLL cells

**Fig. 2:**
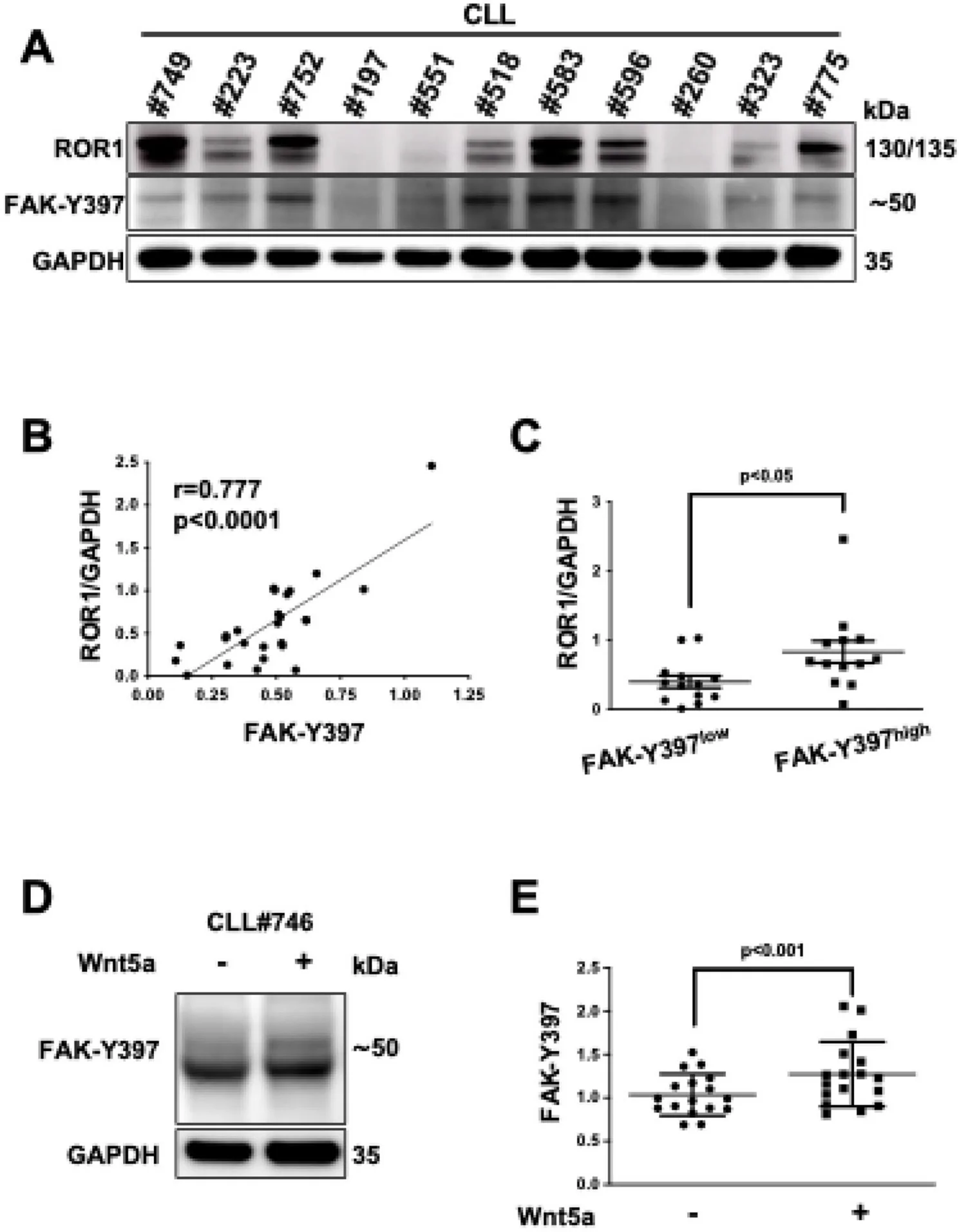
FAK-Y397 activation and its correlation with ROR1 in CLL cells. **(A)** The lysates obtained from leukemic B cells from untreated CLL patients were processed for SDS/PAGE, transferred onto PVDF membrane and immunostained with antibody against FAK-Y397 and ROR1. Membranes were reprobed with anti-GAPDH antibody as loading control. Figure is representative of 11 CLL. **(B)** Data obtained from all the samples (*n* = 26) were analyzed and Pearson’s correlation shows a significant positive correlation between ROR1 and FAK-Y397 expression (*p* < 0.0001). **(C)** FAK-Y397 expression levels obtained have been differentiated using the median FAK-Y397 expression value as a cut-off. ROR1 expression is significantly higher in samples with high FAK-Y397 phosphorylation (FAK-Y397^high^) compared to those with low phosphorylation (FAK-Y397^low^; *p* < 0.05, Mann-Whitney test). **(D)** CLL cells were cultured for 30 min alone or in presence of Wnt5a (200nM). Subsequently, cells were processed for SDS/PAGE, transferred onto PVDF membrane and treated with anti-FAK-Y397 and anti-β-actin antibodies. Figure is representative of 1 sample. **(E)** The total data obtained from the western blots were analyzed and quantification reveals a significant increase in FAK-Y397 phosphorylation following Wnt5a treatment (*p* < 0.001, paired Student’s *t* test; *n* = 17)

Building on our previous findings of a correlation between cortactin/HS1 expression and FAK activation in CLL cells, as revealed by our RPPA analysis, we sought to investigate additional related proteins. Although ROR1 was not included in the RPPA panel, its known variable expression in CLL patients [32] and its overlapping signaling pathways with FAK, particularly involving HS1 and cortactin, provided a strong rationale for examining its role. This decision to study ROR1 was further motivated by its potential involvement in FAK-mediated processes in CLL, complementing our RPPA findings on related proteins. We investigated the correlation between ROR1 and FAK by examining the expression of FAK-Y397 vs. ROR1 in freshly isolated CLL cells that express FAK-Y397 at high levels (FAK-Y397^high^, *n* = 13) vs. low levels (FAK-Y397^low^, *n* = 13) by western blot. We found that the level of ROR1 was significantly higher in FAK-Y397^high^ samples (0.83 ± 0.58) than in FAK-Y397^low^ ones (0.40 ± 0.32; *p* < 0.05, Mann-Whitney test), thus finding a significant correlation between high levels of FAK-Y397 and increased ROR1 expression (*p* < 0.0001, Pearson’s correlation; *n* = 26 - Fig. 2A-B-C). Of note, our preliminary results demonstrated co-localization and co-immunoprecipitation of the two proteins in CLL cell samples (Additional file 1: Fig. S3A-B-C). We also stimulated primary CLL cells (*n* = 17) with exogeneous Wnt5a, the ligand of ROR1, thus finding that this treatment induced a significant increase of FAK phosphorylation at Y397 (1.27 ± 0.37) *versus* the untreated condition (1.04 ± 0.24; *p* < 0.001, paired Student’s *t* test - Fig. 2D-E). ROR1 has been shown to associate with HS1 in CLL cells upon Wnt5a stimulation. [19, 20]. In our cohort, we found HS1 levels to be significantly higher in ROR1^high^ compared to ROR1^low^ cases. (Additional file 1: Fig. S4A-B).

Expanding upon our previous findings linking FAK activation with HS1 (and cortactin) in CLL adverse outcome,^13^ our current results demonstrate that higher levels of FAK activated through Y397 phosphorylation are associated with a potential ROR1/FAK/HS1 axis that merits further investigation as a potential contributor to CLL progression. Moreover, in a small cohort of patients, we correlated the expression levels of ROR1, FAK-Y397, HS1, and µ-calpain (a protease involved in FAK activation),^13^ finding a positive correlation among these proteins. Notably, this increasing trend was predominantly observed in IGHV-unmutated patients (Additional file 1: Fig. S4C-D).

### FAK and ROR1 are overexpressed (and activated) in leukemic cells egressing the lymph nodes

**Fig. 3:**
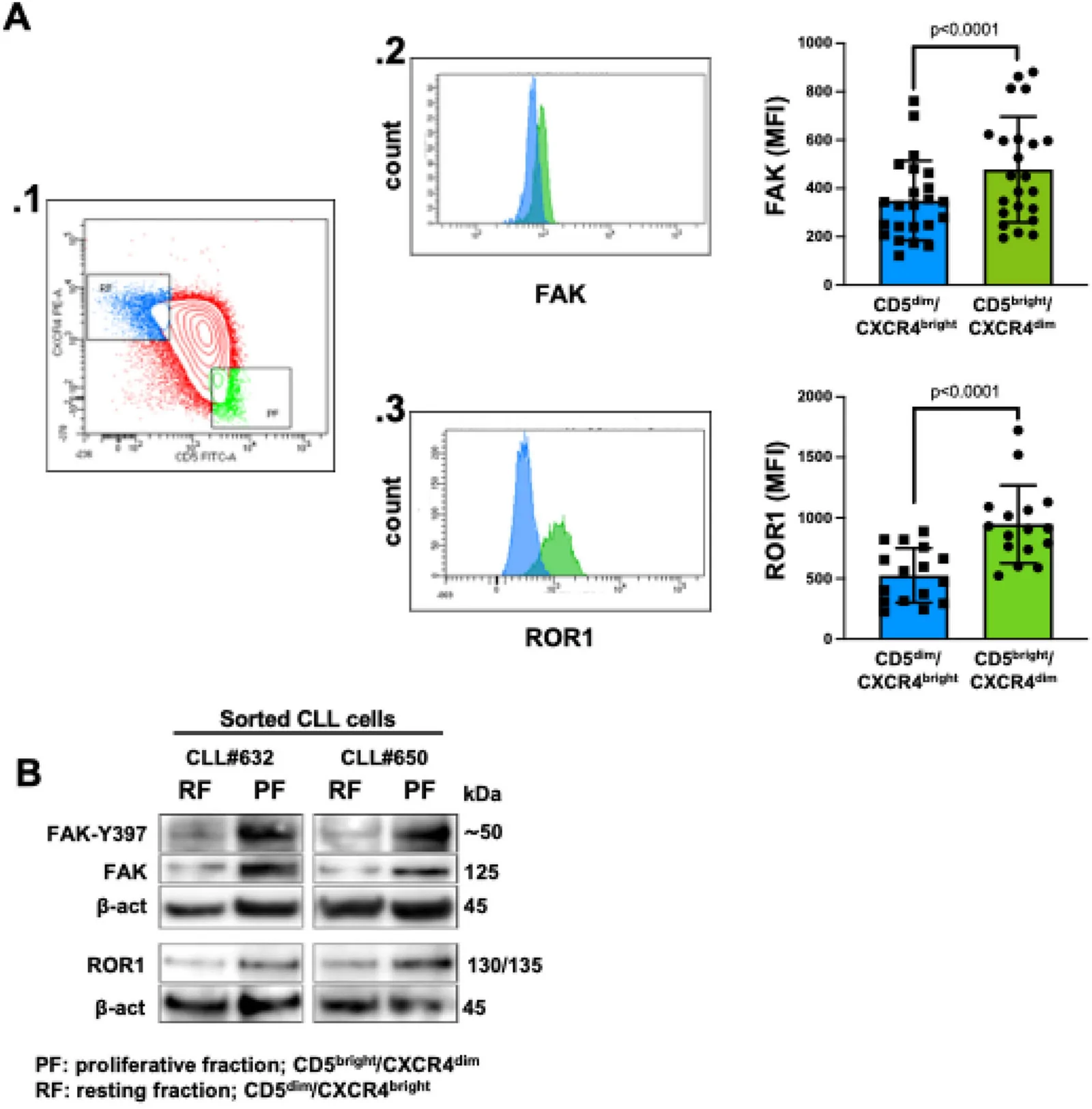
FAK and ROR1 expression in CLL proliferative and resting subpopulations. A1. Freshly isolated CLL cells have been subjected to flow cytometry and analyzed basing on CXCR4 and CD5 expression. Two distinct leukemic subpopulations have been identified: a proliferative fraction (CD5^bright^/CXCR4^dim^, green) and a resting fraction (CD5^dim^/CXCR4^bright^, blue). Contour plot is shown, representing the gating strategy. The expression levels of FAK and ROR1 have been evaluated in the two subsets. **A2.** Intracellular staining revealed significantly higher levels of total FAK in terms of MFI in proliferative compared to resting cells (*p* < 0.0001, paired Student’s *t* test; *n* = 23). **A3.** Surface expression of ROR1 was also significantly higher in the proliferative compared to the resting subset (*p* < 0.0001, paired Student’s *t* test; *n* = 16). **B.** Proliferative and resting fractions of 5 samples analyzed have been physically separated by cell sorting and aliquots have been processed for SDS/PAGE, transferred onto PVDF membrane and immunostained with anti-FAK and anti-ROR1 antibodies. β-actin has been used as loading control. Figure is representative of 2 CLL samples analyzed. MFI= mean fluorescent intensity. PF: proliferative fraction. RF: resting fraction

We analyzed intraclonal FAK and ROR1 expression in different fractions of the leukemic pool for each patient. Based on previous studies [33, 34], we used flow cytometry to identify resting (CD5^dim^/CXCR4^bright^) and lymph node-egressed (CD5^bright^/CXCR4^dim^) populations by their CXCR4 and CD5 expression (Fig. 3A.1). Within these two subpopulations, we then measured the levels of FAK and ROR1. The mean fluorescent intensity (MFI) showed that FAK was significantly more expressed in the fraction of the leukemic clone represented by lymphoid tissues recently egressed cells (477.9 ± 218.5), compared to the older resting fraction (349.3 ± 165.0; *p* < 0.0001, paired Student’s *t* test) that may have been circulating longer in the periphery (Fig. 3A.2). Similar result was obtained for ROR1, that was significantly more expressed in the former fraction (947.5 ± 320.4) compared to the latter (443.0 ± 217.8; *p* < 0.0001, paired Student’s *t* test) (Fig. 3A.3). Of note, this differential expression is stronger for ROR1 in IGHV-unmutated CLL samples (Additional file 1: Fig. S5). We physically separated the two cell fractions using cell sorting, and then investigated FAK, FAK-Y397, and ROR1 expression in each fraction by WB. This analysis confirmed higher expression and activation of FAK and ROR1 in the recently egressed cell fraction (Fig. 3B).

**Fig. 4:**
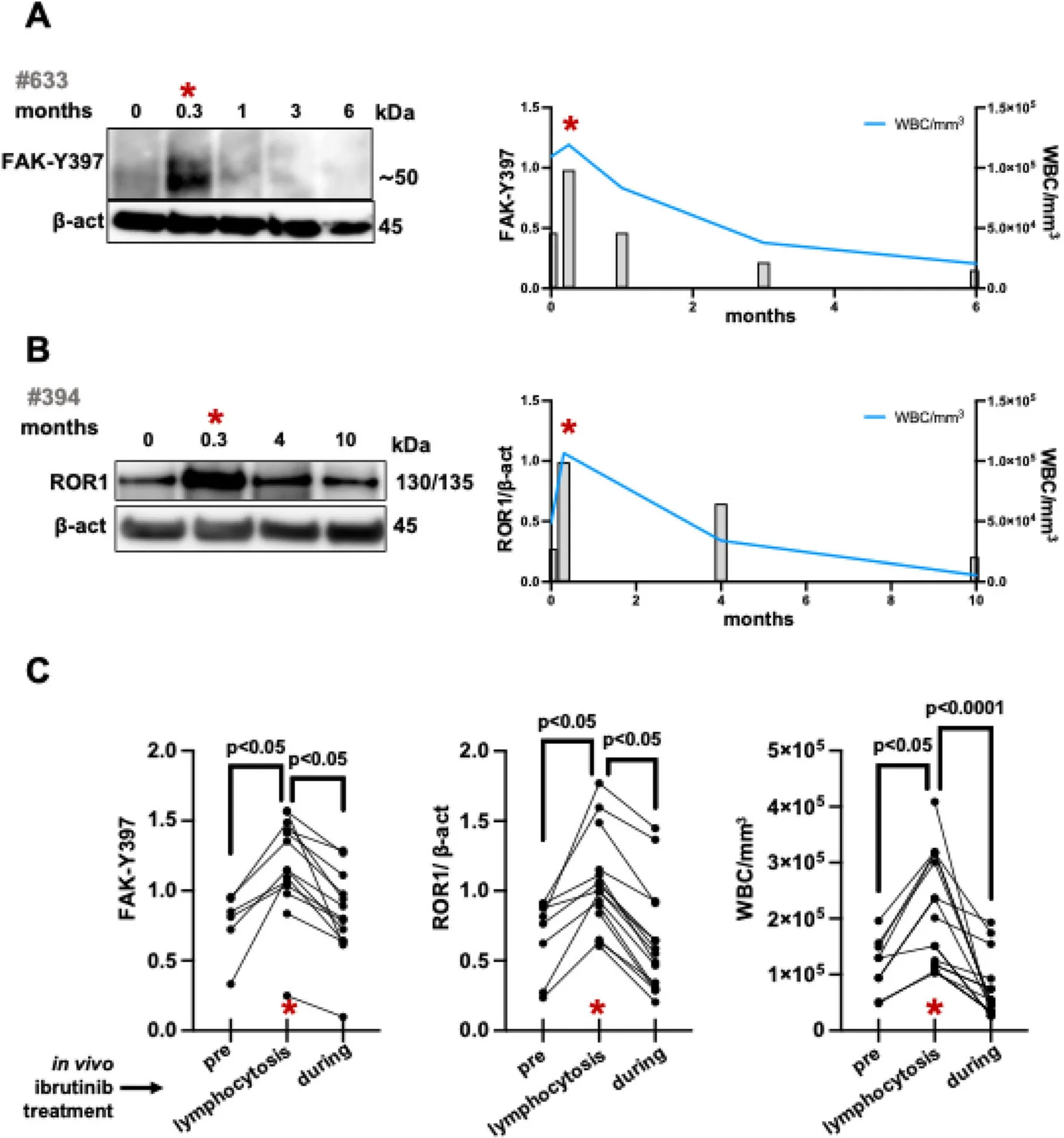
FAK and ROR1 expression in CLL cells during in vivo ibrutinib-containing regimen. **A** and **B.** The lysates obtained from leukemic B cells from CLL patients receiving ibrutinib have been processed for SDS/PAGE, transferred onto PVDF membrane and immunostained with antibody against FAK-Y397 and ROR1, respectively. Different time-points have been taken into consideration. Membranes were reprobed with anti-β-actin antibody as loading control. On the right, protein expression levels of FAK-Y397 and ROR1 for the corresponding patients, including relative densitometric analysis, are shown. The trend of WBC count (WBC/mm^3^, light blue line) is also depicted. **C.** Densitometric analysis of FAK-Y397 and ROR1 protein levels for all the patients analyzed is shown at three different time-points. The figure also reports the corresponding WBC count (right panel), allowing correlation between protein expression dynamics and therapy-induced lymphocytosis. Red asterisks indicate peaks of therapy-induced lymphocytosis. Pre: before ibrutinib administration. During: during treatment and post lymphocytosis

In CLL, the Btk inhibitor ibrutinib characteristically redistributes neoplastic B cells from tissue sites into the peripheral blood, early during therapy. Along with this treatment-related lymphocytosis, enlarged lymph nodes quickly decrease [35, 36]. We analyzed FAK phosphorylation at Y397, as expression of its activation, and ROR1 levels in patients following ibrutinib-containing regimen. Due to ibrutinib mechanism of action, during the first weeks of treatments, patients experienced transient peripheral lymphocytosis (red asterisks). Notably, the mobilized CLL cells displayed increased FAK-Y397 and ROR1 expression. Cells have been analyzed during therapy at different time-points (Fig. 4A and B). In Fig. 4C, data obtained for each patient analyzed with the relative densitometric analysis have been reported. The trend of the white blood cell count (WBC/mm^3^) is also shown. Collectively, these findings point to a possible role of the FAK-ROR1 axis in CLL cell migration and mobilization, which was further explored functionally in the subsequent experiments.

### Defactinib downregulates key survival- and migration-associated proteins in leukemic B cells

**Fig. 5:**
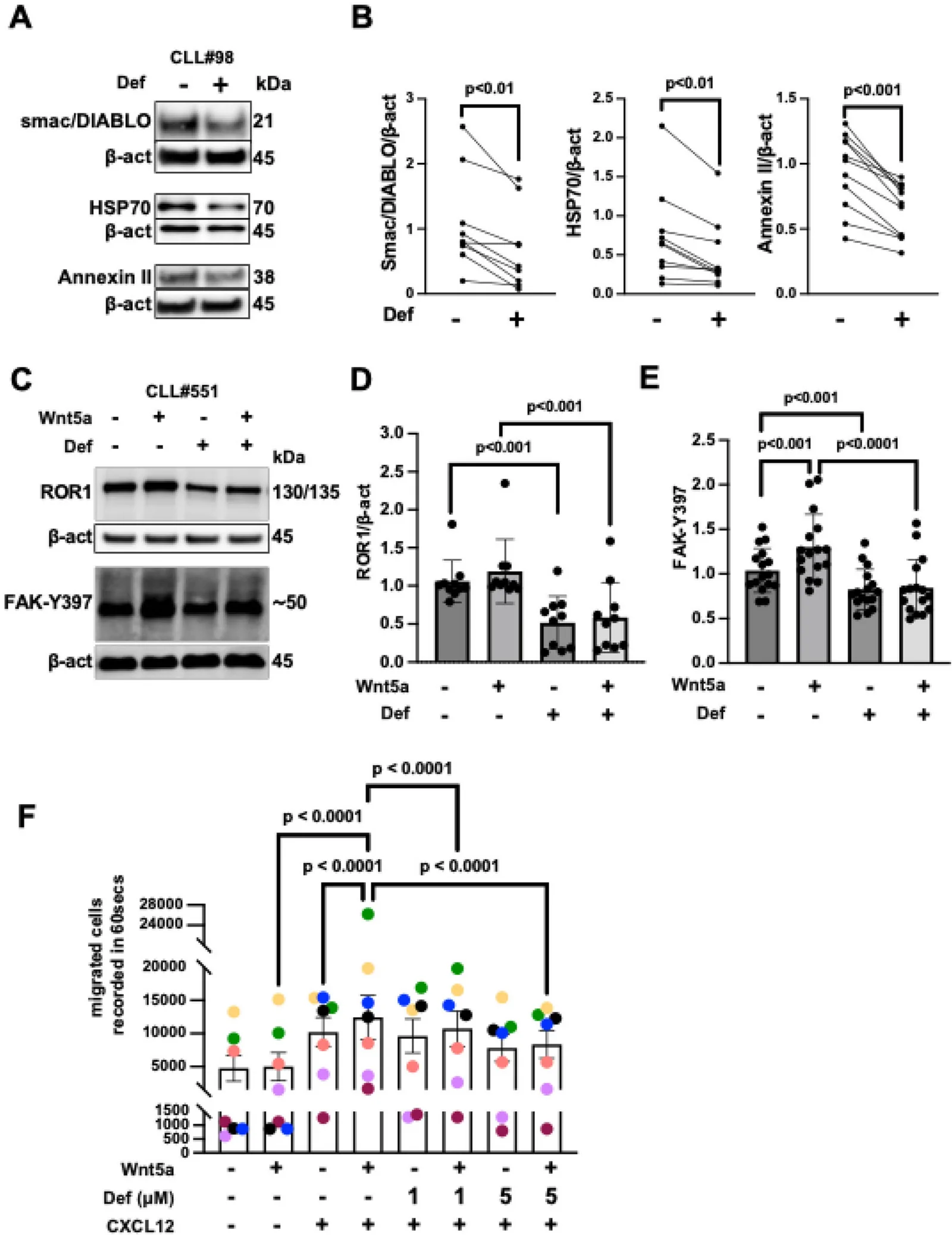
Effects of FAK inhibition on protein expression and chemotactic response in CLL cells.** (A)** CLL cells were cultured for 24 h alone or in presence of defactinib (5µM). Subsequently, cells were processed for SDS/PAGE, transferred onto PVDF membrane and immunostained alternatively with anti-smac/DIABLO, anti-HSP70 and anti-Annexin II antibodies. Membranes have been reprobed with anti-β-actin as loading control. Figure is representative of one sample. **(B)** Graphs represent smac/DIABLO/β-actin, HSP70/β-actin and Annexin II/β-actin ratios of all the samples analyzed (*n* = 9, *n* = 10, *n* = 11, respectively). **(C)** CLL cells were pre-treated or not with Wnt5a (200nM) and then cultured for 24 h alone or in presence of defactinib (5µM). Cells were then processed for SDS/PAGE, transferred onto PVDF membrane and immunostained with anti-FAK-Y397, anti-ROR1 antibodies. Membranes have been reprobed with anti-β-actin as loading control. Figure is representative of one sample analyzed. **(D)** Histograms represent the densitometric analysis of ROR1/β-actin ratio of all the samples analyzed (*n* = 9). **(E)** Histograms represent the densitometric analysis of FAK-Y397 of all the samples analyzed (*n* = 9). **(F)** CLL cell migration in response to CXCL12 (200nM) was measured using a Transwell assay coupled with flow cytometry in patient samples, with or without exogenous Wnt5a (200nM), and in the presence or absence of defactinib (1 and 5µM). Data are reported as mean ± SD of the number of migrated cells aquired in 60 s (*n* = 7). Def: defactinib

We previously demonstrated that defactinib treatment, a FAK inhibitor, caused a decrease of 125 kDa-FAK in leukemic B cells. In addition, FAK activation at Y397 was also reduced in the treated cells [13] Herein, we validated that defactinib is able to attenuate the pro-leukemia phenotype observed in activated-FAK cells (refer to the first paragraph of the results). In particular, after defactinib in vitro treatment we demonstrated a lower amount of Smac/DIABLO in the treated condition (0.67 ± 0.63) compared to the untreated cells (1.09 ± 0.75; *p* < 0.01, paired Student’s *t* test – *n* = 9), of HSP70 (0.48 ± 0.44 vs. 0.73 ± 0.59; *p* < 0.01, Wilcoxon matched-pairs signed rank test – *n* = 10), and of Annexin II (0.65 ± 0.20 vs. 0.94 ± 0.29; *p* < 0.0001, paired Student’s *t* test – *n* = 11) (Fig. 5A-B). We found that defactinib was also capable to significantly reduce ROR1 expression levels (0.52 ± 0.29 vs. 1.06 ± 0.28 of the untreated condition; *p* < 0.001, ANOVA). The same effect of defactinib was observed in cells previously stimulated with Wnt5a (0.59 ± 0.46 vs. 1.12 ± 0.42; *p* < 0.001 - Fig. 5C-D). Moreover, treatment of Wnt5a-stimulated cells with defactinib, reduced the capacity of Wnt5a itself to induce tyrosine phosphorylation of FAK (0.82 ± 0.32 vs. 1.30 ± 0.37 of the untreated condition; *p* < 0.0001 - Fig. 5C-E). To investigate whether FAK is necessary for boosting Wnt5a/CXCL12 chemotaxis, in cells from therapy-free patients we assessed CLL-cell migration using Trans-well assay in response to CXCL12 by flow cytometry, without or with the addition of exogeneous Wnt5a and under conditions whereby active FAK is inhibited by defactinib, at 2 different concentrations, or not. We found that the capability of Wnt5a to enhance chemokine-directed CLL-cell migration is significantly reduced by inhibiting FAK using 1µM (10,725 ± 7,029) as well as 5µM (7,824 ± 5,435) of defactinib compared with the non-inhibited control (12,425 ± 8,738; *p* < 0.0001, ANOVA - Fig. 5F). Overall, these functional data support the ROR1-FAK axis as a contributor to CLL cell mobilization and migration.

### Defactinib boosts the activity of BTK inhibitors

**Fig. 6:**
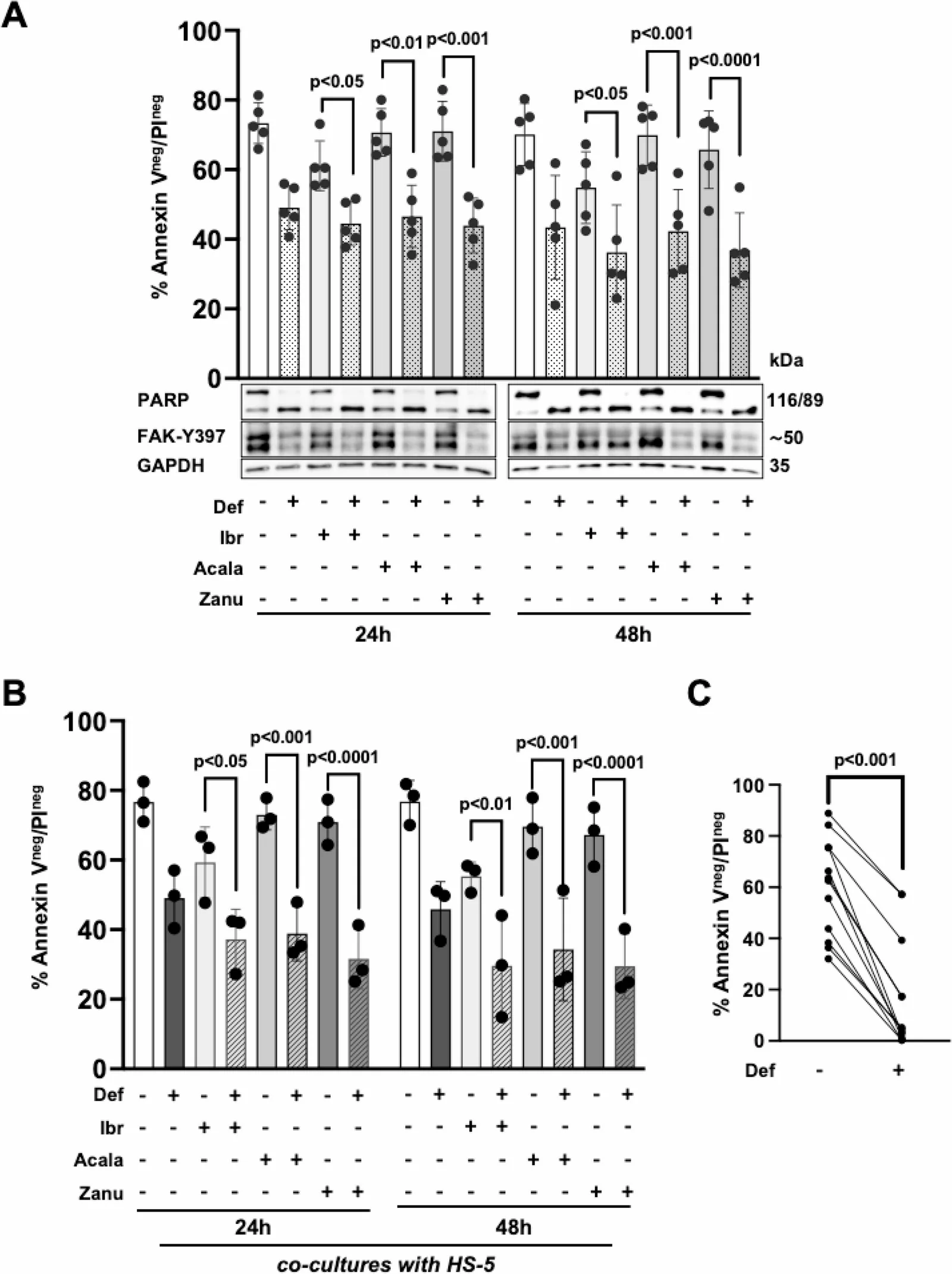
Pro-apoptotic effects of defactinib as a single agent and in combination with BTK inhibitors.** A**. Leukemic **B** cells were cultured for 24 (n = 6) or 48 (n = 5) hours with defactinib (5µM), ibrutinib (5µM), acalabrutinib (5µM), zanubrutinib (5µM), and their combinations. Viability was assessed using the Annexin V/Propidium Iodide assay. Histograms represent the mean ± SD of percentage of cell viability of all the patient’ samples analyzed. The white bars represent untreated control cells (“alone” condition). For all conditions, cells were also processed for SDS/PAGE, transferred onto PVDF membranes and probed with anti-FAK-Y397, anti-PARP, to put in evidence cell apoptosis, and anti-GAPDH as loading control. In the lower panel a representative western blot is shown. **B.** Effect of the same treatments on CLL cell viability in co-culture with HS-5 stromal cells (*n* = 3). Viability was measured after 24 and 48 h using the Annexin V/Propidium Iodide assay. The white bars represent untreated control cells. **C. **Leukemic **B** cells from 12 patients with aggressive disease, including individuals resistant to ibrutinib and those heavily pretreated over several years (Table I) were treated in vitro with defactinib (5 µM) for 24 h, and apoptosis was assessed by flow cytometry. The graph shows the results from Annexin V/Propidium iodide test performed in all the samples. Def: defactinib; Ibr: ibrutinib; Acala: acalabrutinib; Zanu: zanubrutinib

The BTK inhibitor ibrutinib has significantly changed the management of CLL patients achieving high efficacy even in high-risk and chemo-refractory patients [37]. Despite this, relapses occur and the clinical outcome after ibrutinib failure is utmost unfavorable. We and others demonstrated that the FAK inhibitor defactinib was able to induce apoptosis in in vitro CLL cells [12, 13]. We observed the effects of defactinib in combination with ibrutinib and other Btk inhibitors (acalabrutinib and zanutbrutinib) in vitro. Leukemic B cells from 5 CLL patients have been cultured with 5µM defactinib alone or in combination with 5µM ibrutinib, 5µM acalabrutinib or 5µM zanubrutinib and apoptosis has been evaluated after 24 and 48 h. At 24 h time point, CLL cells treated with ibrutinib, acalabrutinib and zanubrutinib showed a viability equal to 61 ± 7%, 70 ± 7% and 71 ± 8%, respectively; cells cultured with drug combinations showed significantly reduced viability to 45 ± 6% (*p* < 0.05), 47 ± 9% (*p* < 0.01) and 44 ± 8% (*p* < 0.001; ANOVA test with respect to BTK inhibitor alone – Fig. 6A-left). At 48 h, the viability of CLL cells treated with ibrutinib, acalabrutinib and zanubrutinib were 55 ± 10%, 69 ± 8% e 71 ± 5%, respectively; cells cultured with drug combinations showed significantly reduced viability to 36 ± 13%, (*p* < 0.05), 42 ± 12% (*p* < 0.001) and 37 ± 11% (*p* < 0.0001; ANOVA test with respect to BTK inhibitor alone - Fig. 6A-right). Protein PARP cleavage analysis by western blotting confirmed an increased apoptosis under co-treatment conditions. A decrease in FAK-Y397 phosphorylation, meaning FAK de-activation, is appreciable, as expected, in all the conditions in which defactinib was present (Fig. 6A, western blotting). Complete flow cytometry data including all Annexin V/PI populations are provided in Additional file 1: Table S2–1.

### Stromal microenvironment does not protect leukemic cells from defactinib-induced apoptosis

CLL cells show prolonged survival in vivo but, isolated from their physiological context and placed in vitro, progressively undergo spontaneous apoptosis. The stroma has in fact a role in the survival of neoplastic B lymphocytes and also in drug resistance [38, 39] We verified defactinib effectiveness in the context of a stromal microenvironment thus co-culturing CLL cells on a stromal layer provided by HS-5 cell line with the previous treatments. At 24 h time point, CLL cells treated with ibrutinib, acalabrutinib and zanubrutinib, on the HS-5 layer, showed a viability equal to 59 ± 10%, 73 ± 4% and 71 ± 6%, respectively; cells co-cultured with HS-5 and drug combinations, presented a viability significantly reduced to 45 ± 9% (*p* < 0.05), 39 ± 8% (*p* < 0.001) and 32 ± 6% (*p* < 0.0001, test ANOVA with respect with BTK inhibitor alone – Fig. 6B-left). After 48 h, in the same co-culture conditions, CLL cells treated with ibrutinib, acalabrutinib and zanubrutinib showed a viability equal to 55 ± 4%, 70 ± 8% and 67 ± 8%, respectively; cells treated with drug combinations presented a viability significantly reduced to 30 ± 15% (*p* < 0.01), 34 ± 15% (*p* < 0.001) and 30 ± 9% (*p* < 0.0001; test ANOVA with respect to BTK inhibitor alone – Fig. 6B-right). Of note, comparisons between defactinib alone and combination treatments in co-culture conditions did not reach statistical significance. Complete flow cytometry data including all Annexin V/PI populations are provided in Additional file 1: Table S2–2. Preliminary data suggest that stromal cells may promote FAK activation in leukemic B cells, potentially contributing to their survival (Additional file 1: Figure S6A). Furthermore, defactinib treatment induced FAK cleavage in HS-5 stromal cells, accompanied by dose-dependent morphological changes including loss of adherent spindle-shaped morphology and detachment from the substrate, without evidence of PARP cleavage, suggesting disruption of FAK-mediated focal adhesions rather than cell death (Additional file 1: Fig. 6B-C).

### Defactinib is effective in Ibrutinib-resistant and in multi-treated CLL patient’s cells

We evaluated defactinib efficacy in inducing apoptosis in leukemic cells from patients with particularly aggressive disease, including those who relapsed on ibrutinib treatment or had heavily pre-treated CLL. Leukemic B cells from 12 such patients (Table 1) were exposed to 5µM defactinib for 24 h. Quantification of apoptosis revealed that defactinib-treated cells showed significantly reduced viability compared to non-treated conditions (17 ± 22% vs. 60 ± 19%, respectively; *p* < 0.001; Wilcoxon matched-pairs signed rank test – Fig. 6C). These results demonstrate defactinib potential effectiveness even in cases of advanced or treatment-resistant CLL.

**Table 1:** Patients resistant to ibrutinib treatment or who have failed multiple lines of therapy

CLL	SEX^1^	AGE (years)	IGHV^2^	FISH^3^	TP53^4^	Th(s) before ibr^5^	Months of ibr	Th after ibr
#223	F	71	M	N	WT	Y	/	zan
#235	M	61	U	12+	WT	Y	12	ven + rit
#253	M	65	U	11q-; 12+	WT	Y	/	zan
#265	F	80	U	13q-	M	Y	31	ven + rit
#403	M	77	U	N	WT	Y	17	ven + rit
#425	M	83	M	N	WT	N	31	acal
#451	M	88	U	12+	nd	Y	12	zan
#610	M	50	M	13q-	WT	N	44	ibr + ven
#611	M	73	U	17p-; 13q-	M	N	16	R/R
#650	M	61	nd	N	WT	Y	72	ven + rit
#761	F	85	U	13q-	M	Y	/	zan
#818	M	65	U	17p-	M	Y	/	zan

^1^M= male; F= female

^2^IGHV mutational status, “Mutated” was defined as having a frequency of mutations greater than 2% from germline VH sequence. M= mutated; U= unmutated; nd= not determined

^3^N= normal karyotype; nd= not determined

^4^WT= wild type; M= mutated; nd= not determined

^5^Y= yes; N= no

Th= therapy; ibr= ibrutinib; ven= venetoclax; zan= zanubrutinib; rit= rituximab; acal= acalabrutinib; R/R= relapsed/refractory

## Discussion

In this study, we observed that Chronic Lymphocytic Leukemia (CLL) samples with higher basal FAK activation were characterized by upregulation of disease-associated pathways, particularly involving ROR1, especially in the IGHV unmutated subgroup. We demonstrated that the focal adhesion kinase (FAK) inhibitor defactinib induces apoptosis in CLL cells, both alone and in combination with BTKi. Notably, defactinib showed efficacy in ibrutinib-resistant or heavily pre-treated patients. These findings suggest FAK, or the FAK-ROR1 axis, as a potential therapeutic target in CLL.

Following stimulation of the BCR, chemokine receptors, and integrins, downstream pathways are activated, contributing to the cancerous phenotype and resistance to therapies [40–44]. These pathways involve the activation of factors such as Btk, PI3K/AKT, NF-kB, actin-binding proteins like HS1 and cortactin, and the release of calcium from intracellular stores in CLL cells. In this context, the non-receptor proline-rich focal adhesion kinase FAK plays a key role in mediating signals from the extracellular environment to the cell interior, ultimately influencing cellular processes such as migration, invasiveness, survival, and proliferation [25].

Our previous research demonstrated a down-modulation of the 125 kDa full-length form of FAK, associated with its calpain-mediated cleavage, following BCR stimulation. In patients with poor prognosis IGHV-unmutated status, we observed a lower amount of full-length FAK, which corresponded to a greater presence of cleaved/activated protein. Notably, we also found that cytoskeletal proteins such as cortactin and HS1, previously implicated in CLL aggressiveness, were overexpressed in those same patients. This observation suggests a possible interaction between these proteins and FAK, potentially contributing to the more aggressive disease phenotype in IGHV-unmutated subgroup [13, 15–17]. Herein, we demonstrated the correlation of activated-FAK (FAK-Y397) with other molecules implicated in CLL aggressiveness, defined by mechanisms of migration, survival, and proliferation. These molecules include Smac/DIABLO, Akt, SHIP1, NF-kB, HSP70, Annexin II, cortactin, HS1 and ROR1.

Our findings suggest a potential link between FAK activation and these pathways that collectively contribute to CLL progression, which is notably more evident in IGHV-unmutated cases. In this context, ROR1 emerges as a key player, sharing connections with the Lyn kinase substrates HS1 and cortactin [19, 20], and being involved in pathways that overlap with those of FAK, as both are implicated in cell migration. Elevated FAK-Y397 levels are notably observed in ROR1^high^ CLL cells and, upon Wnt5a stimulation, FAK phosphorylation at Y397 is promoted, driving its activation. This activation pathway could potentially involve Lyn, highly expressed and activated in CLL [44, 45].

Lyn has been shown to phosphorylate and control ROR1 surface dynamics during chemotaxis of CLL cells [46], and we demonstrated Lyn’s involvement in calcium-dependent calpain activation leading to FAK cleavage and activation in CLL cells [13]. Kipps’ group extensively studied the ROR1 pathway, revealing the roles of HS1 and cortactin in CLL cell migration towards CXCL12 upon Wnt5a stimulation [19, 20]. These findings collectively suggest a potential Lyn-ROR1-FAK signaling axis that could be crucial in CLL cell migration and survival, particularly in IGHV-unmutated cases.

The observed increase in FAK-Y397 following Wnt5a stimulation aligns with calcium mobilization and calpain activation mechanism, highlighting its importance in CLL pathogenesis and, likely, disease progression. Importantly, Lyn kinase, together with its substrates HS1 and cortactin, plays a pivotal role in the crosstalk between the BCR and ROR1 pathways. This intricate network of signaling pathways represents important therapeutic targets in CLL, underscoring the importance of identifying and targeting such crosstalk for developing more effective treatments.

The observation of more prominent FAK activation in patients with 11q deletion, who typically present with lymph node masses, further reinforces FAK’s role in tumor microenvironment interactions. This finding provides a clinical correlation to the molecular observations, emphasizing the potential importance of FAK signaling in the lymph node niche and its contribution to the aggressive phenotype often seen in patients with 11q deletion.

CLL cells circulate in the peripheral blood and accumulate in secondary lymphoid organs. The CXCR4/CXCL12 axis regulates B-cell trafficking and retention, with CXCR4 undergoing enhanced recycling on CLL cells [47]. Calissano et al. identified two CLL subpopulations: CD5^dim^/CXCR4^bright^ cells, a quiescent population resting in the periphery, and CD5^bright^/CXCR4^dim^ cells, a fraction enriched for newly divided cells which are recently egressed from lymphoid tissues [33]. Our flow cytometry analysis revealed significant FAK and ROR1 overexpression in the latter fraction.

While our findings on ROR1 corroborate previous observations by Kipps’ group [48], we demonstrate for the first time a parallel pattern for FAK, collectively supporting their involvement in CLL cell survival and in the phenotype of cells recently egressed from lymph nodes. Similarly, in acute myeloid leukemia (AML), Carter et al. showed that FAK activation by the bone marrow microenvironment promotes leukemia/stroma interactions supporting leukemic cell survival [49]. Taken together, these findings highlight the importance of FAK and microenvironment interactions in leukemia progression.

We recently proved that the treatment with the FAK inhibitor defactinib not only induced apoptosis in CLL cells but also significantly reduced the 125 kDa form of FAK and its phosphorylation at Y397, as well as decreasing HS1 and cortactin expression in CLL cells [13]. In this context, we observed that defactinib also led to a decrease in the expression of some of the above-mentioned disease-associated proteins involved in different survival processes, including HSP70, Annexin II, and Smac/DIABLO. Literature supports the involvement of HSP70 and Annexin II in cell survival and migration mechanisms. Budina-Kolomets et al. showed that HSP70 was overexpressed, particularly in drug-resistant forms of metastatic melanoma, and FAK was identified as an HSP70 “client protein” [50]. We ourselves also demonstrated that HSP70 was overexpressed in CLL cells, according to poor prognosis and drug resistance [26, 28, 51]. The relationship between Annexin II and FAK was documented by Wang et al. in a study on pancreatic cancer, demonstrating that Annexin II activates FAK signaling, observable through phosphorylation at the Y397 activation site, consequently promoting cell proliferation, migration, and invasion [52]. Furthermore, our research revealed that defactinib also decreases the expression of ROR1, even when cells are pre-treated with Wnt5a and, importantly, we observed that Wnt5a-mediated activation of FAK at Y397 is also inhibited by the use of defactinib.

BTKi have been increasingly used to treat various diseases since the FDA first approved ibrutinib, the pioneering BTKi, for mantle cell lymphoma in 2013. Ibrutinib, acalabrutinib, and zanubrutinib are covalent inhibitors that have revolutionized the treatment of CLL. We and others previously demonstrated that defactinib was capable of inducing apoptosis in leukemic B lymphocytes [12, 13]. Herein, we have shown that defactinib, in the same cells, enhances the efficacy of BTKi. Burley et al. had already demonstrated that the synergistic effect of defactinib with ibrutinib was able to induce a significant reduction in cell migration [14]. A significant finding here is defactinib’s ability to induce apoptosis in leukemic cells, either alone or in combination with BTKi, even in the presence of stromal cells. This suggests potential efficacy against protective microenvironmental factors, as stromal cells partially mimic the tumor microenvironment. These results extend our previous findings that defactinib pre-treatment inhibits CXCL12-induced migration of CLL cells [13], now demonstrating its ability to also mitigate Wnt5a-induced migration towards CXCL12. This further emphasize defactinib potential to disrupt key survival and trafficking mechanisms in leukemic cells. Interestingly, preliminary data suggest that the stromal microenvironment may actively promote FAK expression and activation in leukemic B cells, potentially contributing to their survival. Moreover, defactinib was found to directly target FAK in HS-5 stromal cells, inducing FAK cleavage and dose-dependent morphological changes consistent with disruption of FAK-mediated focal adhesions, without evidence of cell death. Taken together, these findings suggest that defactinib may interfere with the supportive capacity of the microenvironment by targeting FAK in both leukemic and stromal cells, thereby potentially disrupting the pro-survival signals delivered to CLL cells within the tumor microenvironmental niche. Clearly, a more in-depth characterization of the effects of defactinib on the stromal compartment would be of great interest and will be the subject of future investigations.

The use of defactinib and BTKi combinations for the treatment of patients with CLL could be hypothesized. Other studies have shown that VS-4718, another FAK inhibitor, was able to prevent cell growth and induce apoptosis in AML cell lines, particularly when combined with ABT-199, a Bcl-2 inhibitor. Similarly, FAK silencing was able to inhibit leukemogenesis in BCR/ABL + ALL cells, increasing apoptosis of tumor cells in combination with imatinib. Furthermore, treatment with VS-4718 in combination with dasatinib was able to inhibit survival in a B-ALL model [53, 54]. Of note, defactinib could prove effective even in those patients resistant or refractory to ibrutinib therapy, as well as in multi-treated ones, as demonstrated by our data. Moreover, considering that defactinib is already being used in clinical trials for some solid tumors, this would potentially reduce the time required for necessary safety and toxicity checks of the drug [55].

The BTKi characteristically redistributes neoplastic B cells from tissue sites into the peripheral blood, early during therapy [35]. We revealed a notable expression of both FAK-Y397 and ROR1 correlating with elevated WBC counts post-ibrutinib in vivo treatment, suggesting FAK’s potential role in facilitating cell redistribution from secondary lymphoid organs to peripheral blood. Further investigations will be crucial to assess FAK-Y397 and ROR1 expression in patients who develop ibrutinib resistance, characterized by lymphadenopathy or increased white blood cell count. Moreover, studies have shown that Wnt5a levels remain elevated in the plasma of ibrutinib-resistant CLL patients [56]. It would be reasonable to hypothesize that, in Ibrutinib-resistant patients, elevated Wnt5a levels could constitutively activate the ROR1 pathway in peripheral blood, potentially resulting also in increased FAK-Y397 levels, thus enhancing cell survival. This may represent an alternative FAK-dependent survival mechanism in ibrutinib-resistant patients who do not harbor BTK mutations, e.g., via AKT or ERK activation. Plasma Wnt5a levels are going to be determined and correlated with FAK-Y397 levels and disease progression.

Our study highlights FAK’s critical role in CLL biology, showing its elevated expression and activation in cells recently egressed from lymphoid tissues, likely promoting cell survival, migration and resistance to targeted therapies.

Consistent with FAK’s known role in cell motility across various cancer types, these findings have significant clinical implications. Patients with high levels of activated FAK may have a more aggressive disease course and could benefit from FAK-targeted therapies, potentially in combination with other treatments like BTKi. While further research is needed to elucidate FAK’s precise mechanisms in CLL progression and explore synergies with other targeted therapies, our findings provide strong evidence that targeting FAK could be a promising strategy to inhibit CLL cell aggressiveness, potentially improving treatment outcomes for CLL patients. ROR1-directed therapies as single agents have demonstrated limited benefit in CLL clinical trials; however, co-targeting FAK and other nodes in this signaling network could enhance therapeutic responses.

## Conclusions

In conclusion, our study identifies FAK as a crucial mediator of CLL pathogenesis, particularly within the IGHV-unmutated subgroup. We provide the first evidence of a functional ROR1-FAK axis that drives leukemia progression. Our findings demonstrate that FAK inhibition via defactinib effectively induces apoptosis, interferes with microenvironmental protection, acts synergistically with BTK inhibitors, and retains efficacy in highly aggressive cases. These results highlight the FAK-ROR1 axis as a high-impact therapeutic target, offering a promising strategy to overcome drug resistance and improve outcomes in high-risk CLL patients.

## Supplementary Information

Below is the link to the electronic supplementary material.

Additional file 1: Supplementary Methods, Figures S1-S6, and Tables S1-S2.

## Data Availability

All data reported in this paper will be shared by F.F. upon reasonable request.

## Abbreviations

BCR: B-Cell antigen Receptor
BTKi: Bruton’s Tyrosine Kinase inhibitors
CLL: Chronic Lymphocytic Leukemia
ERK: Extracellular signal-Regulated Kinase
FAK: Focal Adhesion Kinase
FMO: Fluorescence Minus One
FSC: Forward SCatter
MFI: Mean Fluorescent Intensity
MAPK: Mitogen-Activated Protein Kinase
PBMCs: Peripheral Blood Mononuclear Cells
PI3K: PhosphoiInositide 3-Kinase
RPPA: Reverse Phase Protein Array
ROR1: Receptor tyrosine Kinase-like Orphan Receptor 1
SLO: Secondary Lymphoid Organ
SSC: Side SCatter
