# Supplementary material for: Focal adhesion kinase FAK interplays with ROR1 in the aggressiveness of chronic lymphocytic leukemia
Source: BMC Med. 2026 Jun 27;24:496. doi: 10.1186/s12916-026-05025-1 (PMC13579760; doi:10.1186/s12916-026-05025-1)
Supplement: Supplementary file 1 [file 12916_2026_5025_MOESM1_ESM.docx]

**SUPPLEMENTARY DATA**

**SUPPLEMETARY METHODS**

**Imagestream Analysis**

Imaging flow cytometry was used to measure the expression and colocalization of FAK and ROR1 at the single-cell level. Freshly isolated CLL cells were fixed and permeabilized using the FIX & PERM Cell Permeabilization Kit (Thermo Fisher Scientific; MA, USA). Blocking was performed with PBS1X containing 2% BSA overnight at 4°C. Cells were then stained with anti-FAK mouse monoclonal antibody (Merck KGaA; Darmstadt, Germany) and anti-ROR1 rabbit polyclonal antibody (Cell Signaling Technology CST; MA, USA) at a 1:100 dilution for 1 hour at room temperature in PBS1X with 2% BSA. Secondary staining was done using Alexa Fluor 488 anti-mouse and Alexa Fluor 647 anti-rabbit antibodies (Thermo Fisher Scientific) at a 1:150 dilution for 30 minutes at room temperature. Cells were subsequently stained with 0.1μg/mL DAPI (Merck KGaA) for 2 minutes at room temperature in PBS1X. Controls were performed using the corresponding secondary antibodies alone. Images were acquired at 60X magnification on an ImageStream flow cytometer (DiaSorin; Saluggia, Italy) and analyzed with IDEAS software. Single-color controls were used to create a compensation matrix applied to all files to correct for spectral crosstalk. Colocalization measurements were performed using the BDS feature of IDEAS, with BDS scores greater than 1.25 considered indicative of significant colocalization.

**Immunoprecipitation Analysis**

Direct immunoprecipitation analyses were conducted using the IP Classic Kit (Thermo Fisher Scientific) according to the manufacturer's instructions. Immunoprecipitates were isolated and analyzed by western blotting. Antibodies used included anti-ROR1 (Cell Signaling) and anti-FAK (Merck KGaA).

**SUPPLEMENTARY FIGURES**

**Figure S1.** Patients studied in the RPPA analysis were divided in two groups according to the median value of FAK-Y397 phosphorylation, namely low versus high. Protein expression levels of Smac/DIABLO, AKT-Ser473, NF-κB-Ser536, SHIP-1-Y1020, HSP70, ANNEXIN II, cortactin and HS1 were found to be significantly overexpressed in the FAK-Y397^high^ group.

**Figure S2. Increased phosphorylation of FAK-Y397 in CLL patients with 11q deletion.** **A.** Patients studied in the RPPA analysis^4^ were stratified by their cytogenetic assessed by FISH (13q deletion, normal karyotype, 11q deletion, and 17p deletion) and levels of FAK-Y397 were assessed. Significantly higher levels of activated FAK (phosphorylated at Y397) were found in the 11q– subgroup. **B.** Western blot analysis of selected CLL samples confirms increased FAK-Y397 phosphorylation in patients with 11q deletion. Protein levels were normalized to actin.

**Figure S3.** **Co-localization and co-immunoprecipitation of FAK and ROR1 in CLL B cell. A.** Freshly isolated CLL cells from 5 patients were stained with anti-FAK and anti-ROR1 primary antibodies follow by the appropriate secondary antibodies, *i.e.,* Alexa Fluor 488-conjugated (Green) and Alexa Fluor 647-conjugated (Red) respectively, and cells were acquired in the ImageStream instrument. Bright Detail Similarity (BDS) scores were calculated to quantify colocalization between FAK and ROR1 signals. Representative cells with colocalized signals (BDS scores 1.25) are shown. **B.** ImageStream histograms illustrate colocalization patterns of ROR1 and FAK in CLL cells, indicating weak (*e.g.*, CLL #427 and #799) and strong (*e.g.*, CLL #752, #765, #98) colocalization based on BDS analysis. The % of co-localization is reported. **C.** Freshly isolated B cells from one patient were processed to immunprecipitate FAK and the lysate obtained from immunoprecipitated-FAK fraction was processed for SDS/PAGE, transferred on nitrocellulose membrane and immunostained with antibody against ROR1 and FAK, as control (left). Total cell lysate obtained from the same CLL patient was processed for SDS/PAGE, transferred on nitrocellulose membrane and immunostained with antibody against FAK and ROR1 to assess the presence of both proteins in the sample and anti-β-actin was used as loading control (right).

**Figure S4. Correlation of FAK-Y397/ROR1/HS1/μ-calpain in CLL patients. A. The lysates obtained from leukemic B cells from CLL patients** have been processed for SDS/PAGE, transferred on nitrocellulose or PVDF membrane and immunostained **with antibody against ROR1 and HS1. GAPDH has been used as loading control.** ROR1 expression levels obtained have been differentiated using the median ROR1 expression value as a cut-off. HS1 expression was significantly higher in samples with high ROR1 (ROR1^high^, n= 9) compared to those with ROR1 low levels (ROR1^low^, n= 10; p<0.001, Mann-Whitney test). **B.** Data obtained from all the samples (n= 18) were analyzed and Pearson’s correlation shows a significant positive correlation between ROR1 and HS1 expression (p<0.0001). **C.** In a set of 10 CLL samples, the lysates obtained from leukemic B cells from CLL patients have been processed for SDS/PAGE, transferred on nitrocellulose or PVDF membrane and immunostained with antibody against ROR1, FAK-Y397, HS1 and μ-calpain. **GAPDH has been used as loading control. Values from densitometric analysis were plotted in a heat map. IGHV genes mutational status is also shown. M= mutated. U= unmutated. (D) ROR1 expression has been evaluated in leukemic B cells form CLL patients (n= 5 IGHV-mutated and n= 5 IGHV-unmutated) by flow cytometry.**

**Figure S5. FAK and ROR1 expression in proliferative and resting subpopulations from mutated- and unmutated-IGHV CLL patients. Referred to figure 3Ai,** **freshly isolated CLL cells have been subjected to flow cytometry and analyzed basing on CXCR4 and CD5 expression. Two distinct leukemic subpopulations have been identified: a proliferative fraction (CD5^bright^/CXCR4^dim^, green) and a resting fraction (CD5^dim^/CXCR4^bright^, blue). Total samples (n= 23 for FAK and n= 16 for ROR1 staining) have been divided by meaning of the presence or not of somatic hypermutations in IGHV genes. Samples from patients where the mutational status of IGHV genes was not available were not included in the analysis.**

**Figure S6. Effect of the stromal microenvironment on FAK in CLL cells and impact of defactinib on HS-5 stromal cells. A. Representative Western blot analysis of full-length FAK (fl-FAK), FAK phosphorylation at Y397 (FAK-Y397), PARP cleavage, and β-actin (loading control) in CLL cells cultured in the presence (+) or absence (-) of bone marrow stromal cells (BMSC) from CLL patients for 24h. Dot plots show quantification of fl-FAK/β-actin and FAK-Y397 levels across individual patient samples (n=5). *p<0.05. B. Representative Western blot analysis of PARP, full-length FAK (fl-FAK), cleaved FAK (cl-FAK, indicated by the blue arrows), and β-actin in HS-5 stromal cells treated with increasing concentrations of defactinib (0, 5, 10 µM) for 24h and 48h. C. Bright-field microscopy images of HS-5 stromal cells treated with defactinib (0, 5, 10 µM) for 48h, showing dose-dependent morphological changes including progressive loss of adherent spindle-shaped morphology and detachment from the substrate.**

**SUPPLEMENTARY TABLES**

**TABLE S1. Clinical characteristics of CLL patients present in the study.**

**^1^**M= male; F= female

**^2^**IGHV mutational status, “Mutated” was defined as having a frequency of mutations greater than 2% from germline VH sequence. M= mutated; U= unmutated; nd= not determinable, that is: direct Sanger sequence analysis did not allow the identification of a clonal rearrangement or the assessment of the IGHV mutational status; na= not available

**^3^**N= normal karyotype; na= not available

**^4^**WT= wild type; M= mutated; na= not available

**^5^**Th(s)= previous treatment history. Y= yes; N= no. All patients were treatment-free for at least 6 months prior to the experiment. For patients included in the longitudinal analysis (Figure 4C), samples were collected at three time points: "pre" (treatment-free or off therapy for at least 6 months), "lymphocytosis" and "during" (patients were receiving ibrutinib-based treatment regimens)

**TABLE S2. Annexin V/PI staining data for Figures 6A and 6B. Distribution of CLL cell populations across treatment conditions at 24h (section 1) and 48h (section 2), without or with HS-5 co-culture.**

**1.**

**2.**
